# Sarcoglycanopathies: From clinical diagnosis to new promising therapies

**DOI:** 10.1177/22143602251324855

**Published:** 2025-03-25

**Authors:** Holly Borland, Jordi Diaz-Manera

**Affiliations:** 1 The John Walton Muscular Dystrophy Research Center, Newcastle University Translational and Clinical Research Institute, Newcastle upon Tyne, UK; 2 Genetics department, Newcastle Upon Tyne Hospitals Trust, UK

**Keywords:** sarcoglycanopathy, limb girdle muscular dystrophy, gene therapy, phenotype, genotype

## Abstract

The sarcoglycanopathies are a severe form of limb girdle muscular dystrophy caused by mutations in the sarcoglycan genes SGCA, SGCB, SGCG, and SGCD, leading to reduced or absent expression of the alpha-, beta-, gamma-, and delta-sarcoglycan proteins respectively. Most patients develop a severe disease starting in the first decade of life that progresses quickly and eventually leads to a loss of ambulation before the age of 20. However, there is a marked heterogeneity in the prognosis, and several patients develop a milder phenotype. The factors correlating with disease progression are not completely known, but recent data suggest that remaining protein expression can be a key factor. The diagnosis is confirmed by genetic studies, which are sometimes not confirmative in the case of identifying variants of unknown significance or just one variant. New tools to understand the potential pathogenesis of missense variants have been developed; these are helping in the diagnosis of these diseases. Additionally, recent data on muscle MRI have revealed a characteristic pattern of involvement that can also support the diagnosis of the disease. In recent years, data coming from international collaborative studies have allowed an understanding of disease progression; however, this is only through retrospective data. There are no prospective studies collecting longitudinal data on skeletal and respiratory muscle function or cardiac structure and function progression over time which is hampering the development of new drugs in the field. Clinical trials with gene therapy are underway or are being designed in some of the subtypes of sarcoglycanopathies to advance therapeutic management.

## Introduction

The sarcoglycanopathies are a type of limb-girdle muscular dystrophy (LGMD) caused by pathogenic variants in the SGCA, SGCB, SGCG and SGCD genes, encoding the alpha-, beta-, gamma-, and delta-sarcoglycan proteins respectively.^1^ These proteins are expressed in the muscle membrane of the skeletal muscle fibres.^1^ Epsilon and zeta sarcoglycan genes also encode proteins present at the skeletal muscle membrane; however, they are not linked to any muscular dystrophy.^2,3^ The former four sarcoglycan proteins (i.e., alpha, beta, gamma, and delta) assemble as a complex in the endoplasmic reticulum and subsequently become associated with other proteins to form the dystrophin-associated glycoprotein complex (DGC).^4^ The DGC forms a strong connection between the intracellular cytoskeleton of the muscle fibre and the extracellular matrix, contributing to structural integrity and signal transduction of striated skeletal and cardiac muscles.^4^ Within the DGC, the sarcoglycans contribute specifically to membrane permeability and membrane integrity, preventing degradation.^4^ Therefore, the reduced or absent expression of such proteins within the complex results in increased membrane fragility and decreased strength and integrity, leading to muscle fibre damage and degeneration.^4–8^ Consequently, mutations in the SGCA, SGCB, SGCG, and SGCD genes are responsible for a LGMD type: 2D/R3, 2E/R4, 2C/R5, and 2F/R6 respectively.^1^ There is epidemiological variation in the global distribution of the different subtypes (Table 1): while LGMDR3 is relatively common in Europe and the US, LGMDR4 is most common in the Iranian population, and LGMDR5 is most common in Indian, Algerian, Tunisian, and gypsy populations.^9–16^ Sarcoglycanopathies usually present with slowly progressive muscle weakness during childhood; however, there is great variation in the phenotypical presentation among patients.^9,13,17–19^ Moreover, there is significant heterogeneity within each subtype, ranging from asymptomatic or mild forms to severe forms involving limited ambulation and cardiac and respiratory implications.^20^ Retrospective studies have suggested that the amount of remaining protein expression influences disease progression.^5,13,20–24^ Null mutations lead to absent protein expression, and patients harbouring such mutations usually present in early childhood and have a severe clinical picture. Conversely, missense mutations lead to reduced, but not absent, protein expression. Although these retrospective data are available, there is a need for good quality prospective data on disease progression to confirm whether there are differences between each subtype to help improve care provision and clinical trial design for these diseases. There are no standardised guidelines for the diagnosis and care of patients with sarcoglycanopathies, representing one of the unmet needs for these patients. There are two pharmaceutical companies planning clinical trials with gene delivery strategies using adeno-associated virus (AAV) for these diseases. In this review, we aim to summarize the information available about clinical presentation, diagnosis, disease progression and care, and discuss new therapies arriving to the field. We will highlight what are, in our opinion, the unmet needs in sarcoglycanopathies that require further research.

**Table 1.** Main clinical, genetic, and epidemiological data of the different subtypes of sarcoglycanopathy.

	Alpha-Sarcoglycanopathy (LGMDR3)	Beta-Sarcoglycanopathy (LGMDR4)	Gamma-Sarcoglycanopathy (LGMDR5)	Delta-Sarcoglycanopathy (LGMDR6)
Age at onset	Childhood or adulthood	Childhood, often before age 8 years	Childhood, often before age 5 years	Childhood, often before 10 years
Clinical Severity	Variable phenotype From mild to severe	Rapid, severe disease progression	Rapid, severe disease progression	Severe phenotype
Early Clinical Features	Asymptomatic hyperCKaemia Exercise intolerance Myalgia and/or rhabdomyolysis Proximal symmetrical lower limb weakness Tip toe walking Calf hypertrophy Tendon contractures	HyperCKaemia Exercise intolerance Myalgia and/or rhabdomyolysis Muscle cramps Proximal symmetric muscle weakness Limb and trunk muscle atrophy Calf hypertrophy – tip toe walking	HyperCKaemia Exercise intolerance Tiptoe walking Scapular winging Calf hypertrophy Muscle weakness and wasting	HyperCKaemia Exercise intolerance Proximal muscle weakness Distal lower limb muscle weakness Gait disturbance Scapular winging Calf hypertrophy
Late Clinical Features	Loss of ambulation Respiratory impairment Joint contractures Scoliosis	Loss of ambulation Cardiac impairment Respiratory impairment Joint contractures Scoliosis	Loss of ambulation Severe cardiac impairment Respiratory impairment Joint contractures Scoliosis	Loss of ambulation Scoliosis Cardiac impairment Respiratory impairment
Genetic Features	Mostly compound heterozygous variants Majority are missense variants Nonsense variants are rarer, giving a more severe phenotype	Majority are missense variants Nonsense variants are rarer, giving a more severe phenotype	Homozygous variants are predominant. Majority are missense variants Nonsense variants are rarer, giving a more severe phenotype	Homozygous variants Frameshift, nonsense, and splicing variants reported Most pathogenic variants affect the extracellular domain of the protein
Epidemiological Distribution	Widely distributed	Highly prevalent in North Europe and Iran and in populations with high prevalence of consanguinity	Widely distributed, but high frequency in India and Maghreb countries, especially Algeria and Tunisia Romany Gypsies of Europe	Ultrarare disease: families described in Brazil, Iran, and India

## General clinical phenotype

Although the specific clinical phenotypes depend on subtype, the sarcoglycanopathies typically present in childhood with weakness of the proximal muscles of the shoulder and pelvic girdles^17,19^ (Table 1). In the lower limbs, there is an early involvement of the glutei and adductor muscles, followed later by involvement of the quadriceps. In the upper limbs, deltoid, triceps and biceps are affected in almost all patients, as well as periscapular muscles leading to frequent bilateral scapular winging.^25^ Distal muscle involvement is less prevalent but may occur in more advanced stages and in severe disease, while hand and facial muscles tend to be spared.^20,25^ Tiptoe walking, difficulty climbing stairs, positive Gower's sign, and calf and tongue hypertrophy are often seen early in disease progression.^17,19,20^ Axial muscle weakness progresses over time, leading to scoliosis. Most patients will lose ambulation and develop cardio-respiratory involvement during the second decade of life.^26^ However, there is variation in disease progression ranging from severe Duchenne-like muscular dystrophy to milder phenotypes characterised by difficulties climbing stairs or rising from floor.^27^ Patients with mild disease usually present after ten years of age and experience slow disease progression.^9^ Occasionally, individuals carrying pathogenic variants in these genes are entirely asymptomatic, with isolated hyperCKaemia, or they present with exercise-induced myalgia and myoglobinuria.^13,20,27–29^ Respiratory muscle weakness is often seen in patients later in disease progression when patients have already lost ambulation. Respiratory involvement might require patients to use nocturnal non-invasive ventilation (NIV); some patients require daytime use as well. Cardiac manifestations in the form of arrhythmias and dilated cardiomyopathy may be present in all subtypes.^20,30^ Such manifestations are most frequent and severe in beta- and delta-sarcoglycanopathy, possibly due to the expression of beta- and delta-sarcoglycan in coronary vessels, leading to added coronary injury.^20,30^ Cardiac impairment may necessitate the use of cardioprotective medications, pacemaker implementation, and cardiac transplantation.^30–32^

### Alpha-sarcoglycanopathy

Alpha-sarcoglycanopathy is the most common form of sarcoglycanopathy in Europe.^16^ Clinically, it tends to be the mildest of the sarcoglycanopathies.^19–21,27–29^ Despite its tendency to present as the most mild form, alpha-sarcoglycanopathy actually has the most variable phenotype among the sarcoglycanopathies, with presentations ranging from asymptomatic hyperCKaemia to severe Duchenne-like disease involving loss of ambulation and respiratory impairment.^20,22,25^ All patients have some degree of skeletal muscle degeneration as indicated by elevated creatinine kinase (CK) values.^25^

### Beta-sarcoglycanopathy

Beta-sarcoglycanopathy accounts for 15–30% of all sarcoglycanopathies.^20,32^ It appears to be the most common form of sarcoglycanopathy in the Iranian population.^12^ Moreover, there appears to be a higher frequency of disease in populations with a high degree of consanguinity.^20,33^ The mild form of beta-sarcoglycanopathy is relatively rare and may remain as isolated presentations of myoglobinuria or rhabdomyolysis before ultimately developing clinical features.^34,35^ The common form is more rapid and severe.^19,20,34^ Due to the ubiquitous presence of beta-sarcoglycan in cardiac muscle, cardiac involvement is also a frequent feature, affecting more than 50% of patients.^20,30,32,36^

### Gamma-sarcoglycanopathy

Gamma sarcoglycanopathy is the second most frequent form of sarcoglycanopathy in many European and South American countries, behind alpha sarcoglycanopathy.^20^ It is also very prominent in Maghrebian countries and India.^20^ Conversely to alpha sarcoglycanopathy, mutations in the gamma-sarcoglycan gene tend to cause a severe Duchenne-like phenotype.^16,20^ Severe muscle wasting, respiratory impairment, dilated cardiomyopathy, and premature death are common features.^16,20,37,38^

### Delta-sarcoglycanopathy

Compared to the other three subtypes of sarcoglycanopathy, mutations in the delta-sarcoglycan gene are extremely rare.^20,39^ This subtype of the disease tends to manifest in populations with a family history and/or a high degree of consanguinity but does not appear to be highly concentrated in any particular geographical location.^20,39^ Delta-sarcoglycanopathy manifests in a similar manner as the other subtypes; proximal muscle weakness and cardiac involvement is common.^39^ It has been theorised that the incredibly low prevalence of the disease may be attributed to cardiac failure during foetal development, resulting in miscarriage or stillbirth.^39^ However, this theory has not been supported with evidence. Respiratory compromise requiring ventilatory support is less common than cardiac impairment but may occur in up to a quarter of cases.^39^

## Genotype-phenotype correlations

Disease severity depends on the type of mutation and the degree of residual protein. Nonsense and frameshift mutations disrupt the reading frame or cause a shortening of the transcript so are incompatible with protein production.^21^ Such mutations therefore result in a more severe phenotype, characterised by early age at onset, faster disease progression, early loss of ambulation, and shorter life expectancy.^5,20–22,34^ Conversely, missense mutations and those that maintain the reading frame allow for production of a protein that will be processed at the endoplasmic reticulum before reaching the membrane. At this point, misfolded dysfunctional proteins can be identified and eliminated by the endoplasmic reticulum-associated protein degradation system while others can escape and reach the membrane effectively. Therefore, it can be difficult to predict protein expression in missense mutations.^20–23^ However, remaining protein expression is associated with a milder phenotype. The degree of residual protein expression is inversely related to disease severity: the greater the degree of expression, the milder the disease.^13,20,21^ This remains true for all subtypes of sarcoglycanopathy.^5,21,22,24,34^ Despite overlap between the subtypes, there are significant phenotypic differences depending on the specific sarcoglycan subtype gene mutation. In general, alpha- and gamma-sarcoglycanopathies tend to be milder, whereas beta- and delta-sarcoglycanopathies are more severe.^21,23^

## Diagnostic investigations

### Clinical assessment

A diagnosis of sarcoglycanopathy should be considered in all patients with proximal muscle weakness of a suspected inherited origin. Differential diagnoses include X-linked Duchenne Muscular Dystrophy (DMD), Becker Muscular Dystrophy (BMD), other childhood-onset LGMDs (e.g., calpainopathy), metabolic myopathies with myoglobinuria, and idiopathic hyperCKaemia.^20^ A thorough clinical assessment should be conducted initially to gain insight into the patient's motor development; ability to rise from floor; presence of spinal rigidity, scoliosis, and contractures; and ability to run, hop, and jump.^19^ Clinicians should pay particular attention to the presence of certain typical features of LGMD to distinguish between the different types. In the case of sarcoglycanopathies, these include presentation in the first or second decade of life with proximal muscle weakness; muscle hypertrophy, particularly of the calves and tongue; muscle weakness affecting glutei muscles early that progresses quickly to affect proximal upper and lower limb muscles; scapular winging; and, later in the progression of the disease, development of Achilles tendon contracture and respiratory and cardiac involvement.^19^ Rate of progression and disease severity varies among individuals. However, there is a predilection to certain features depending on the subtype, as described previously. Following clinical assessment, initial investigations could include genetic analysis, muscle imaging (i.e., magnetic resonance imaging (MRI)), muscle biopsy, and laboratory investigations.

### Genetic analysis

Genetic analysis providing confirmation of the mutated gene is the gold standard for diagnosis. Next generation sequencing (NGS) can study all the involved genes simultaneously to confirm the diagnosis by distinguishing between milder cases of dystrophinopathies, as well as identifying subtle or elusive mutations.^20,40–42^ Protein investigation via muscle biopsy is useful in determining the pathogenicity of variants of unknown significance (VUS). Recent publications offer a guide to predict the pathogenesis of missense variant in the sarcoglycan genes.^43^ If pathogenic variants are found via NGS, diagnosis can be confirmed, and management can be initiated. If a VUS or just one variant is elucidated, MRI and biopsy are recommended as avenues for further investigation. Genetic analysis remains a challenge due to the various types of mutations, so it is important that a combination of genetic analysis and muscle biopsy is used for accurate diagnosis.

### Laboratory investigations

Measurement of serum CK levels is a simple and effective indicator of muscle damage. CK levels are almost definitely raised, usually over five times the upper limit of normal, indicating muscle deterioration and damage.^19,20^ Although helpful in supporting a diagnosis of a muscle disease, elevated CK levels alone are not specific to sarcoglycanopathy and are therefore not sufficient to confirm a diagnosis.^19^

### Muscle imaging

**Figure 1.**
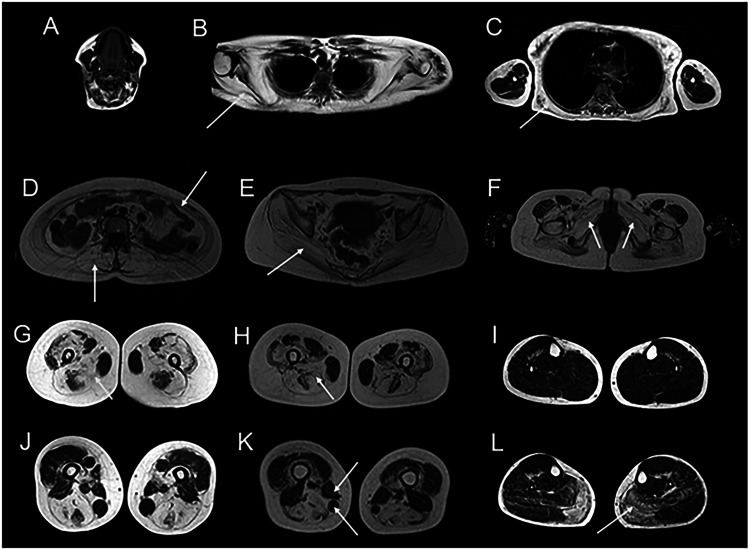
Findings on muscle MRI studies in sarcoglycanopathies.

Muscle imaging, including MRI and computed tomography (CT) scan, helps clinicians determine patterns of muscle involvement and assess severity.^19^ In the case of sarcoglycanopathies, early changes are visualised in the adductor muscles of the pelvic girdle, specifically in adductor magnus. As the disease progresses, there is typically symmetrical involvement of the muscles of the pelvic girdle, with the adductor magnus and glutei muscles most frequently and severely affected.^11^ On MRI, this is indicated by complete or almost complete fat infiltration as seen in Figure 1. The smaller pelvic and obturator muscles; biceps femoris; and proximal vastus intermedius, medialis, and lateralis are often affected with disease progression.^11^ Involvement of the distal lower leg muscles in the sarcoglycanopathies is rare and typically only occurs with loss of ambulation. Even in the most advanced patients with severe, widespread muscle involvement, there appears to be sparing of flexor digitorum longus and tibialis posterior of the lower limbs.^11^ If lower leg involvement is present early in the disease, consideration of an alternative diagnosis may be warranted.^11^

A shows no involvement of cranial muscles. B shows severe fat replacement of periscapular muscles (arrow). C shows involvement of serratus anterior muscle which is associated to scapular winging. D shows severe fat replacement of abdominal and paraspinal muscles (arrows). E shows involvement of all glutei muscles (arrows). F shows severe involvement of the pelvic floor muscles (arrows). In the thigh, there is severe involvement of anterior and posterior muscles (G, H, J, K). However, a gradient of quadriceps involvement, with the proximal section being more involved than distal, is commonly observed in patients as shown in two patients (G and J, H and K). Adductor major muscle is commonly affected (arrow in G and H). Sartorius and gracilis tend not to be involved, even in late stages of the disease (arrow in K). I shows no involvement of the leg muscles, while L shows involvement of gastrocnemius medialis and soleus (arrow).

### Muscle biopsy

**Figure 2.**
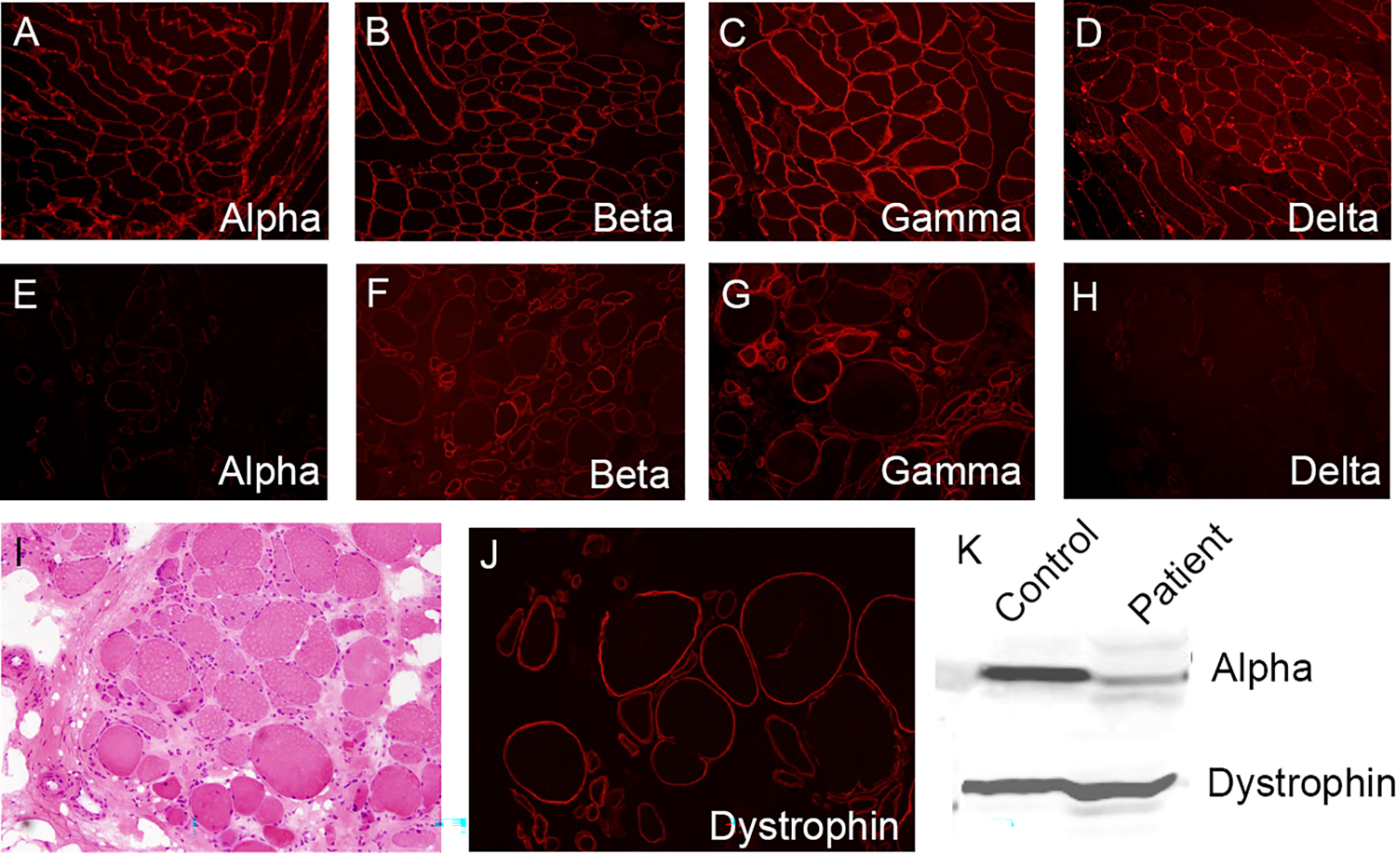
Expression of sarcoglycan proteins in the muscle fibres of patients with a genetically confirmed alpha-sarcoglycanopathy.

Muscle biopsy with histological analysis of the clinically affected muscle is indicated in those where results of genetic analysis are uncertain. Results from muscle imaging (i.e., muscle MRI or CT) can help guide the site for biopsy through identification of a clinically affected muscle that is not completely replaced by fat.^19^ Standard histological techniques on the sample will reveal dystrophic features present in all LGMDs, including variation in fibre size, increased proportion of central nuclei, endomysial fibrosis, necrosis, and regeneration.^19,20^ Occasionally, mild myopathic changes may also be present.^13,19,20^ In all cases of sarcoglycanopathy, there will be variable reduction of expression of one or all of the four sarcoglycan proteins (i.e., alpha, beta, delta, or gamma) in the sarcolemma as seen in Figure 2.^13,44^ In most cases, this results in deficiency of the whole sarcoglycan subcomplex.^13,44^ Immunohistochemistry should be performed with all four anti-sarcoglycan antibodies; results of this will likely show single or multiple abnormalities.^19^ As of yet, there are no immunohistochemical staining patterns specific to the sarcoglycanopathies. However, research indicates there is often an absence of all four sarcoglycans in beta- and delta-sarcoglycanopathy, whereas gamma-sarcoglycanopathy usually shows an absence of only gamma-sarcoglycan.^20^ Analysis of sarcoglycan proteins is sensitive in detecting the primary sarcoglycanopathies.^20^ However, it is not specific, as other muscular dystrophies may cause secondary reduction of sarcoglycan expression.^20^ Staining for dystrophin is also essential to rule out diagnoses of DMD and BMD.^19^ Protein expression can also be quantified using western blot, which can be useful to predict patients’ severity, as remaining expression higher than 30% has been linked to milder disease with a better prognosis.^20^

All four sarcoglycans were studied in muscle biopsies of controls and a patients with a genetically confirmed alpha-sarcoglycanopathy. A, B, C and D show normal expression of alpha, beta, gamma and delta sarcoglycan while E, F, G and H show reduced or absent expression of the same proteins. I shows typical dystrophic pattern in an haematoxylin-eosin staining. J shows reduced expression of dystrophin in several fibres when staining was performed. H shows reduced alpha-sarcoglycan expression and normal dystrophin expression studied by Western-Blot.

## Management

### General approach

Management of patients with sarcoglycanopathy should involve a multidisciplinary approach to address all aspects of care and ensure the best quality of life. There are no treatments specific to sarcoglycanopathy. In general, a supportive and palliative approach should be taken, tailored to the individual and the subtype. Overall goals include maintenance of muscle strength, joint range of movement, and respiratory and cardiovascular function.^44^

### Physiotherapy, orthoses, and exercise

Physiotherapy is essential in maintaining or improving muscle strength, maximising functional ability, and minimising the development of contractures for all patients.^45^ Physiotherapy should be initiated immediately following diagnosis. In an effort to ensure compliance, it is important to implement exercises that the patient and their family find to be beneficial without being too overwhelming or burdensome.^45^ Hydrotherapy is an effective form of therapy for children; however, access may be limited.^45^ Additional interventions include supportive tools, such as knee-ankle-foot orthoses (KAFO), to be used at bedtime in an effort to prevent contractures.^45^ Should scoliosis or joint contractures develop, surgery may be required to correct the deformity for functionality.^46^ Strength and moderate aerobic exercise may be beneficial.^45–47^ However, exercise must be approached with caution due to the risk of exercise-induced myoglobinuria or rhabdomyolysis with high-intensity training.^46,47^ Therefore, clinicians should encourage patients to perform low-impact exercise, hydrate adequately, and advise on warning signs of overexertion.^47^

### Steroid use

Few studies have reported benefit with use of corticosteroids (i.e., deflazacort and/or prednisolone) showing improvement or stabilisation of muscle strength.^46,48–50^ Moreover, research suggests that steroids may be beneficial in the cardiac management of patients with muscular dystrophies.^31,51,52^ Within the sarcoglycanopathies, some studies suggest that deflazacort or prednisolone may improve left ventricular ejection fraction or stabilise cardiac function.^51^ However, limited evidence means their use is not yet indicated for muscle symptoms or cardiac involvement in patients with sarcoglycanopathy.^46,51^

### Respiratory management

Progressive muscle wasting often results in respiratory impairment, warranting coordination with respiratory physicians who have experience in neuromuscular diseases. Frequent monitoring of respiratory function via forced vital capacity (FVC) is essential from diagnosis. When values drop below 60% predicted, overnight pulse oximetry monitoring is recommended to guide decisions on intervention.^19^ Non-invasive positive-pressure ventilation (NIV) should be initiated when night-time hypoventilation is detected by pulse oximetry or FVC declines below 50%.^19,53^ Studies illustrate that NIV can improve survival and quality of life in patients with muscular dystrophies.^54^ Chest physiotherapy and cough assist devices are also recommended in all muscular dystrophies with respiratory involvement to help effectively clear airways, improve peak cough flow, and prevent infections.^53,54^ General measures including annual influenza vaccination and early treatment of respiratory infections is also essential.^53,54^

### Cardiac management

Development of dilated cardiomyopathy is a serious concern in patients with sarcoglycanopathy, so close cardiac surveillance in conjunction with a cardiologist is required from diagnosis.^53^ Monitoring with echocardiography and electrocardiography should occur at least annually, especially after loss of ambulation, as most patients are asymptomatic initially.^20,46^ If cardiac impairment is detected by abnormal echocardiography findings, symptomatic treatment should be initiated. This includes use of diuretics, angiotensin-converting enzyme (ACE) inhibitors, and beta-blockers.^20,46,53^ The need for cardiac transplantation is rare but may be indicated if cardiac failure progresses despite optimal management.^20,46,53^

## Emerging therapies and future

### Gene therapy

As with many of the other genetic forms of muscular dystrophy, gene therapy is being investigated as a major treatment option for the sarcoglycanopathies. Gene therapy using adeno-associated virus (AAV) vectors is one of the preferred methods due to the low immunogenicity, lack of genomic integration, and high success rates in pre-clinical and clinical trials in a variety of muscular dystrophies.^44^ Pre-clinical trials have used AAV1 and AAV8 serotypes; both have shown positive effects.^55–57^ Results from these studies indicate that targeted AAV-mediated transfer of the sarcoglycan genes in knockout mice provides expression of the desired protein at the muscle sarcolemma, improved histopathology of the targeted muscles, and increased force generation and motor function.^44,55–61^ Such results have been illustrated in all subtypes of sarcoglycanopathy. Moreover, AAV transfer in young knockout mice has shown to have prophylactic effects, preventing the onset and progression of dystrophic changes.^60,61^ In pre-clinical studies examining the safety and efficacy of systemic AAV-mediated sarcoglycan delivery, results indicate improvement in histopathology and functionality of both skeletal and cardiac muscle.^57,62^

Human clinical trials of gene transfer with AAV vectors have also shown promising results. Although limited in number and sample size, phase I/II trials have indicated that targeted gene transfer of alpha- or gamma-sarcoglycan via an AAV1 vector results in increased gene and protein expression.^55,56,63^ Robust protein expression seems to be maintained for 3–6 months following targeted muscle injection.^55,56^ The overall safety of gene therapy in human trials has been favourable, aside from two cases of treatment-related elevated gamma-glutamyl transferase (GGT) levels.^55,56,63^ There have been no reported adverse immune responses.^55,56,63^

Preliminary results have been published from an ongoing phase 1/2 study by Mendell et al. investigating the use of gene therapy with systemic delivery of bidridistrogene xeboparvovec for beta-sarcoglycanopathy.^63^ Results indicate that bidridistrogene xeboparvovec has a favourable safety profile overall, with no adverse immune responses toward the vector or beta-sarcoglycan protein itself. Moreover, immunofluorescence staining suggests robust SGCB expression at 60 days. There was also sustained improvement at 2 years in functional outcome measures, as indicated by results on the North Star Assessment for Limb-girdle Type Muscular Dystrophies (NSAD) and timed function tests. This study is ongoing with final results expected to be published at five years. The overall safety of gene therapy in human trials has been favourable, aside from two cases of treatment-related elevated gamma-glutamyl transferase (GGT) levels.^55,56,63^ There have been no reported adverse immune responses.^55,56,63^

Additional phase 1/2 studies investigating the use of gene therapy for patients with sarcoglycanopathy are in the pipeline and will soon start recruiting patients with sarcoglycanopathy (NCT05973630, NCT06747273). These studies involve systemic administration of sarcoglycan genes via AAV vectors.

### Pharmacotherapeutic approaches

Although experimentation with gene therapy is the focus of clinical trials for the sarcoglycanopathies, alternative pharmacological approaches for management are being explored. The primary source of fibrosis in muscular dystrophies is due to abnormal activity of fibroblasts and fibroadipogenic progenitor cells (FAPs), so drugs that target signalling of these cells to disrupt fibrosis are being researched. Nintedanib, a tyrosine kinase inhibitor (TKI) which inhibits such signalling, has been shown to reduce muscle fibrosis, necrosis, and inflammation, as well as improve motor function in mouse models of alpha-sarcoglycanopathy.^64^ In one study, Nintedanib also had favourable outcomes from a cardiac perspective, with improved systolic function following administration.^64^

The P2X7 subtype of the P2 purinergic receptors is part of another proinflammatory pathway that usually triggers inflammation in response to ATP.^65^ In dystrophic muscle, there is overexpression of the P2X7 receptor and high levels of extracellular ATP, resulting in increased P2X7 activity.^66–69^ Therefore, drugs antagonising the P2X7 receptor are being investigated. Results from murine studies in alpha-sarcoglycanopathy indicate that knockout mice treated with a P2X7 antagonist had improved histological, biochemical, and functional endpoints.^67^ It has been suggested that drugs targeting both of the aforementioned proinflammatory pathways (i.e., Nintedanib and P2X7 antagonists) could be used alone or in combination with gene therapies to preserve muscle architecture.^64,70^

Although only currently licensed for use in diabetes mellitus and polycystic ovarian syndrome, metformin is another drug that is being investigated in the management of sarcoglycanopathies. Studies indicate that metformin can have cardioprotective effects in patients with sarcoglycan-related cardiomyopathy, resulting in reduced fibrosis, reduced hypertrophy, and less degeneration.^71^ Systemically, in dystrophic mice, it has been shown to improve muscle function and prevent muscle damage through stimulation of AMP-activated protein kinase (AMPK).^72,73^ Studies also indicate that metformin induces a reduction in biomarkers indicative of muscle degradation and remodelling, such as fibrotic cytokines, matrix metalloproteinases (MMP), CK, and lactate dehydrogenase (LDH).^73^

Finally, cystic fibrosis transmembrane conductance regulator (CFTR) correctors are being investigated for use in the sarcoglycanopathies. Cystic fibrosis (CF) and sarcoglycanopathy have very similar pathogenic mechanisms, so theories suggest that compounds able to recover mutated CFTR would be active on sarcoglycan genes too. Evidence from pre-clinical trials indicates that CFTR correctors can recover mutants of sarcoglycan genes defective in folding and trafficking and reroute the sarcoglycan complex to the sarcolemma.^74–77^ This results in increased protein expression, improved localisation to the sarcolemma, and improved muscle function.^74–77^

## Conclusion

Despite continuous research and growing knowledge about the sarcoglycanopathies, a lot remains unknown due to the rarity of the disease. Retrospective studies have provided information about the apparent phenotypical presentation of the disease; however, little is known about the natural history and progression due a lack of longitudinal trials. Longitudinal studies, such as JOURNEY (NCT04475926), are ongoing to ascertain more information about the natural history of the disease and provide insights for clinical trial development and implementation. Moreover, standards of care guidelines are being developed for global implementation to ensure equitable management of patients, as well as lay a foundation for comparator groups in clinical trials.
